# The Mechanism of Enterohepatic Circulation in the Formation of Gallstone Disease

**DOI:** 10.1007/s00232-014-9715-3

**Published:** 2014-08-09

**Authors:** Jian-Shan Cai, Jin-Hong Chen

**Affiliations:** Department of General Surgery, Huashan Hospital, Fudan University, 12 Wulumuqi Road, Shanghai, 200040 People’s Republic of China

**Keywords:** Bile acids metabolism, Enterohepatic circulation, Transporters, Nuclear receptor, Gallstone formation

## Abstract

Bile acids entering into enterohepatic circulating are primary acids synthesized from cholesterol in hepatocyte. They are secreted actively across canalicular membrane and carried in bile to gallbladder, where they are concentrated during digestion. About 95 % BAs are actively taken up from the lumen of terminal ileum efficiently, leaving only approximately 5 % (or approximately 0.5 g/d) in colon, and a fraction of bile acids are passively reabsorbed after a series of modifications in the human large intestine including deconjugation and oxidation of hydroxy groups. Bile salts hydrolysis and hydroxy group dehydrogenation reactions are performed by a broad spectrum of intestinal anaerobic bacteria. Next, hepatocyte reabsorbs bile acids from sinusoidal blood, which are carried to liver through portal vein via a series of transporters. Bile acids (BAs) transporters are critical for maintenance of the enterohepatic BAs circulation, where BAs exert their multiple physiological functions including stimulation of bile flow, intestinal absorption of lipophilic nutrients, solubilization, and excretion of cholesterol. Tight regulation of BA transporters via nuclear receptors (NRs) is necessary to maintain proper BA homeostasis. In conclusion, disturbances of enterohepatic circulation may account for pathogenesis of gallstones diseases, including BAs transporters and their regulatory NRs and the metabolism of intestinal bacterias, etc.

## Introduction

Gallstone disease is a frequent and economically relevant health problem worldwide. In Western countries, the morbidity of cholelithiasis is about 10–20 % (Kratzer 1999). Moreover, between 20 and 40 % of gallstone patients become symptomatic or develop complications (Gibney 1990), and more than 700,000 people in the US and 170,000 in Germany underwent cholecystectomies annually (Sandler et al. 2002). Economically, gallstone disease has been identified as the second most costly disorder of the digestive tract. A genetic component in the susceptibility to cholesterol gallstones has been recognized (Zubler et al. 1998). Bile acid pool size is maintained relatively constant at about 3–5 g in healthy subjects by two mechanisms, enterohepatic circulation and de novo synthesis of bile acids. This latter mechanism compensates for the daily facal loss (about 0.2–0.6 g) of bile acids, whereas the majority of the pool is conserved by the former mechanism (Lanzini and Lanzarotto 2000). The term enterohepatic circulation (EHC) denotes the movement of bile acid molecules from the liver to the small intestine and back to the liver. Bile acids traverse the hepatocyte and are actively secreted into canalicular bile, completing the enterohepatic cycle. During the enterohepatic circulation, bile salts encounter populations of facultative and anaerobic bacteria, which is relatively small in quantity but rather diversified in the small bowel. Bile salt metabolized by small bowel microbes consists mainly of deconjugation and hydroxy group oxidation. Ileal bile salt transport is highly efficient (about 95 %), but approximately 400–800 mg of bile salts escapes the enterohepatic circulation daily and becomes substrate for significant microbial biotransforming reactions in the large bowel (Ridlon et al. 2006).

## Bile Acid Biotransformation by Bacteria

### Bile Salt Hydrolase(s)

The human liver can produce close to 1 L of bile every day, but comparatively small amounts are lost from the body. Hence, approximately 95 % of the bile acids distributed to the duodenum are reabsorbed into venous blood within the ileum and colon, and subsequently, through mesenteric vein, they arrive at the portal vein, finally they approach the sinusoids of the liver. And hepatocytes re-uptake the bile acids capably from sinusoidal blood, while small amounts escape into systemic circulation. Bile acids are resecreted into canaliculi by the hepatocyte afterward. As a whole, the enterohepatic circulation makes each bile salt molecule available several times during a solitary digestive stage. During these processes, an important biotransformation that must take place before subsequent modifications is termed deconjugation (Batta et al. 1990), catalyzed by the bile salt hydrolase (BSH).

BSH is an enzyme produced by several bacterial species in the human or animal gastrointestinal tract that catalyzes the glycine- or taurine-linked bile salt deconjugation reaction. And it belongs to the choloylglycine hydrolase enzymes family, which also comprises penicillin amidases. Both of them have been classified as an N-terminal nucleophilic (Ntn) hydrolase with an N-terminal cysteine residue. BSH catalyzes the hydrolysis of amide bond in the conjugated bile salts (CBS), then forms the deconjugated bile acid (mainly cholic and quenodeoxycholic) until free amino acids are dissociated. These primary bile acids may afterward undergo dehydroxylation and get converted into secondary bile acids (deoxycholic and lithocholic) after a series of changes.

BSH has already been identified that its substrate (bile acids) is either on amino acid groups (glycine/taurine) or on cholate steroid nucleus. There have been a number of reports on cholate group identification by BSH. It has been reported in several literatures that among the BSHs whose substrate is at amino acid moieties, the hydrolysis of glycoconjugated bile salts are usually more efficient than the tauroconjugated bile salts (Oh et al. 2008; Kim et al. 2004; Tanaka et al. 2000). A *Lactobacillus buchneri* JCM1069 exhibited hydrolase activity against the taurodeoxycholic acid but not against the taurocholic acid, although both acids had taurine as their amino acid moiety, they varied in their steroid moieties at 7α position (Moser and Savage 2001).

Some of probiotics with BSH have been recognized and characterized. Interestingly bile salt tolerance has generally been considered more important than that of the other properties during probiotic selection in *Bifidobacterium*, such as gastric and pancreatic tolerance. It has also been observed that pancreatin supported *Bifidobacterium* to survive in the gastrointestinal tract and withstand against the antimicrobial property of bile acids (Masco et al. 2007). It is possible that the presence of BSH and some transporter proteins lead to bile salt tolerance of some microorganisms, which are functionally related to the response efficiently to the stress from bile salts (Kim and Lee 2008). Microbial traits must be well tolerant to bile acid if they are to survive in the human gut (Dethlefsen and McFall-Ngai 2007). Jones et al. (2008) hypothesized that BSH facilitates colonization by mediating the resistance to the conjugated bile acids.

BSHs are very specific for certain bile types and they help bacteria to survive in various bile environments through their contact with bile. This assumption is supported by the studies carried out in *Lactobacillus plantarum* WCFS1 that have four BSH genes, and *L. acidophilus* NCFM that have two BSH genes (McAuliffe 2005; Bron et al. 2006). Bile addition may sometimes have inducing or inhibiting effects on BSHs as they are highly substrate specific. Researches have shown that the expression of BSH 1 by the bile was induced sixfold, while that of BSH 3 was reduced fivefold. Two BSH genes of *L. acidophilus* NCFM were inactivated, which indicated that the encoded enzymes possessed different substrate specificities (Bron et al. 2006). It is also known that different parts of bile stimulate different BSHs. BSH A activity was stimulated by the steroid nucleus of bile salts, while the activity of BSH B was induced by the amino acid side chain (McAuliffe 2005).

BSH activity has been found mostly in Gram-positive commensals (except a few Bacteroides), which also acquire genome homolog, whereas it is lacking in Gram-negative commensals of the gastrointestinal tract. *Escherichia coli* and *Salmonella enterica* serovar typhimurium are reported as BSH-negative strains (Begley 2005). The prevalence of BSH is well recognized among the established probiotic genera. It is observed foremost in the majority of species such as genera *Lactobacillus* (McAuliffe 2005), *Bifidobacterium* (Kim and Lee 2008), *Bacteroides* (Kawamoto et al. 1989), and *Enterococcus* (Franz et al. 2001). Tanaka et al. (1999) screened more than 300 strains from *Bifidobacterium*, *Lactobacillus*, *Lactococcus lactis*, *Leuconostoc mesenteroides*, and *Streptococcus thermophilus* and found BSH activity in 273 strains in *Bifidobacterium* and *Lactobacillus* but missing in *L. lactis*, *L. mesenteroides*, and *S. thermophilus*. Furthermore, Tannock et al. (1989) conclude that lactobacilli are the main contributors to total BSH activity in the murine intestinal tract, according to the fact that BSH activity in the ileal of these mice was reduced by 86 % in the absence of lactobacilli and by greater than 98 % in the absence of lactobacilli and enterococci compared with samples from control group mice.

Microbial genome analyses have identified homologs and putative BSH genes recently. It varies from species and genera in the organization and regulation of genes encoding BSH. Monocistronic BSH genes have been reported in *La. plantarum* (Christiaens^1992^), *La. johnsonii* (Elkins et al. 2001), *Li. monocytogenes* (Dussurget et al. 2002), and *Bi. bifidum* (Kim et al. 2004). And the crystal structure showed that the enzyme encoded by the CBAH-1 gene forms an active homotetramer (Rossocha et al. 2005). Polycistronic operons have been characterized that three genes are involved in bile salt deconjugation (cbsT1, cbsT2, and cbsHb), which are detected in *La. Johnsonii* and *La. acidophilus* (Elkins et al. 2001). Genes cbsT1 and cbsT2 appear to be gene duplications that encode taurocholate/CA antiport proteins of the major facilitator superfamily, however, cbsHb encodes the BSH b-isoform (Elkins and Savage 2003). BSH expression is also growth phase-dependent. Stationary phase expression has been reported in *Bacteroides fragilis* (Stellwag and Hylemon 1976), and exponential phase expression was reported for *Bi. longum* (Tanaka et al. 2000).

### Bile Acid Hydroxysteroid Dehydrogenase (HSDH)

The initial step in bile salt transformation is deconjugation of taurine- and glycine-conjugated bile salts to the respective unconjugated free bile salts. This process is mentioned previous. Free bile salts are further processed via reductive dehydroxylation. Oxidation/reduction of hydroxy groups at C-3, C-7, and C-12, epimerization of hydroxyl groups, and 7α/β-dehydroxylation, these processes generate the so-called secondary bile salts deoxycholate and lithocholate, which are produced from the primary bile salts cholate and chenodeoxycholate, respectively. HSDHs are nicotinamide adenine dinucleotide phosphate (NADPH)-dependent enzymes belonging to the short-chain dehydrogenase/reductase (SDR) superfamily (Kavanagh et al. 2008). They catalyze the oxidation/reduction of hydroxyl groups of neutral steroids, bile acids, and other steroid derivatives.

Oxidation and epimerization of the 3-, 7-, and 12- hydroxy groups of bile acids in the GI tract are carried out by HSDH expressed by intestinal bacteria. Epimerization of bile acid hydroxy groups is the reversible change in stereochemistry from α to β configuration with the generation of a stable oxo-bile acid intermediate. Epimerization requires the concerted effort of two position-specific, stereochemically distinct HSDHs of intraspecies or interspecies origin. For example, the presence of both 7α and 7β-HSDH in *C. absonum* allows epimerization by a single bacterium (Sutherland and Macdonald 1982), whereas epimerization also can be achieved in cocultures of intestinal bacteria, one possessing 7α-HSDH and the other 7β-HSDH (Hirano and Masuda 1981; MacDonald et al. 1982). The extent of the reversible oxidation and reduction of bile acid hydroxy groups by HSDH depends in part on the redox potential of the environment. Addition of oxygen to the culture medium increases the accumulation of oxo-bile acids (Sutherland and Macdonald 1982).

#### 3α- and 3β-HSDHs

3α-HSDHs specifically catalyze the reversible, stereospecific oxidation/reduction between 3-oxo-bile acids and 3α-hydroxy bile acids. 3α-HSDHs have been detected in some of the most prevalent intestinal bacteria, including *C. perfringens* (Macdonald et al. 1976), *Peptostreptococcus productus* (Edenharder et al. 1989), and *Eggerthella lenta* (formerly *Eubacterium lentum*) (MacDonald et al. 1979, 1977), as well as in intestinal bacteria present in lower numbers (<105/g wet weight of feces), including *C. scindens* (Mallonee et al. 1995) and *C. hiranonis* (Wells and Hylemon 2000), and in nonintestinal bacteria, including *Pseudomonas testosteroni* (Skalhegg 1975). This reversible process is catalyzed by NAD(P)-dependent enzymes related to the short-chain dehydrogenase/reductase superfamily (Hoffmann and Maser 2007).

3β-HSDHs specifically catalyze the reversible, stereospecific oxidation/reduction between 3-oxo-bile acids and 3β-hydroxy bile acids. 3β-HSDHs activities have been described in species of *Clostridium* and *Rumminococcus*. And 3β-HSDHs have also been proved to preferentially require NADP(H), with the exception of *C. innocuum*, which uses NAD(H) (Edenharder et al. 1989). Dihydroxy bile acids [deoxycholic acid (DCA), chenodeoxycholic acid (CDCA), and ursodeoxycholic acid (UDCA)] are generally better substrates than trihydroxy bile acids [cholic acid (CA)] (Edenharder et al. 1989; Macdonald et al. 1983). In fact, three copies of 3α-HSDH genes (the bile acid-inducible genes baiA1, baiA2, and baiA3) have been identified from *C. scindens* (Gopal-Srivastava 1990; Coleman et al. 1988), and baiA1 has been expressed in *E*. *coli* and characterized (Mallonee et al. 1995).

#### 7α- and 7β-HSDHs

Specifically, 7α-HSDHs and 7β-HSDHs might be utilized for the selective α/β inversion of the hydroxy group at C-7 of primary bile acids, which is identified at an industrial level through a multistep chemical process (Iida and Nishida 1993). Both enzymatic activities have been detected in many intestinal bacteria (Lepercq et al. 2004). Genes encoding for 7α-HSDHs have been cloned from *E. coli* (Yoshimoto et al. 1991), *B*. *fragilis* (Bennett et al. 2003), *Clostridium sordellii* (Coleman et al. 1994), and *Eubacterium* sp (Baron et al. 1991) strains. Moreover, the crystal structure of the *E. coli* 7α-HSDH was determined (Tanaka et al. 1996), and not only the role of active site residues in catalysis but also the cofactor recognition were investigated. 7α-HSDHs generally use NADP(H) as a cofactor, with the exception of *E. coli* (Prabha and Gupta 1989) and *Ba. thetaiotaomicron* (Sherrod and Hylemon 1977). *C. bifermentans*, *C. absonum*, and *Ba. fragilis* 7α-HSDHs that use either NAD(H) or NADP(H) as a cofactor (Macdonald and Sutherland 1983; Hylemon and Sherrod 1975; Sutherland et al. 1987) On the contrary, less information is available concerning 7β-HSDHs, enzymes showing this activity has been partially purified from *Ruminococcus* sp (Akao et al. 1987). and *Peptostreptococcus productus* (Edenharder et al. 1989). Recently, the gene encoding the NADPH-dependent 7β-HSDH from *Collinsella aerofaciens* was identified and cloned (Liu and Aigner 2011).

#### 12α- and 12β-HSDHs

The epimerization of the 12α/β hydroxyl group by microbial cooperation was demonstrated. Previous research shows that the enzyme catalyses the oxidation of CA directly to 12-oxochenodeoxycholic acid. Human intestinal flora exist cooperating microorganisms such as *Clostridium* group P strain C 48-50, expressing NADP(H)-dependent 12α-hydroxysteroid dehydrogenase (HSDH) (Macdonald et al. 1979), and *Clostridium paraputrijiicum*, strain D 762-06, containing a described NADP(H)-dependent 12P-HSDH (Edenharder and Pfutzner 1988). NADP-dependent 12α-HSDHs have been detected in *Bifidobacterium* species (Aries 1970) and *C. leptum* (Harris and Hylemon 1978) in *Clostridium* group P (Macdonald et al. 1979), whereas NAD-dependent 12α-HSDH activity was reported in *Eg. lentum* (MacDonald et al. 1977) and *C. perfringens* (Macdonald et al. 1976). Meanwhile 12β-HSDHs have been detected in *C. Tertium*, *C. Difficile*, and *C. paraputrificum* (Edenharder and Pfutzner 1988; Edenharder and Schneider 1985). So far, *Clostridium* group P, strain C 48–50, is the only microorganism known to express HSDH activity at unusually high level, meanwhile in the absence of other HSDHs (Macdonald et al. 1979). Potentially, this microorganism is a good source for producing HSDH. 12α/β-HSDHs characterized to date are constitutively expressed and noninducible, with the exception of the 12β-HSDH from *C. paraputrificum*, which is induced by 12-oxo-bile acid substrates (Edenharder and Pfutzner 1988). 12α/β-HSDHs generally have higher affinity for dihydroxy bile acids (DCA) than for trihydroxy bile acids (CA and iso-CA) and for free versus conjugated bile acids. The 12α-HSDH from *C. leptum* is an exception, demonstrating higher affinity for CA conjugates than for free CA (Harris and Hylemon 1978).

### 7α/β Dehydroxylation of Bile Acid

Owing to the fact that 7-dehydroxylation cannot be reversed by the host enzymatic machinery, LCA and DCA tend to accumulate in the BA pool. However, LCA is 3-sulfated and conjugated at C-24 by the liver, resulting in a derivative that is poorly absorbed from the colonic mucosa, and consequently LCA is not present in significant amounts in the bile (Hofmann 2004). Thus major BAs in human bile are CA, CDCA, and DCA, which are accompanied by minor amounts of UDCA, LCA, and other BAs, whereas faces contain mainly DCA, LCA, along with minor amounts of CDCA, CA and UDCA ,and a variety of bacteria transformed derivatives (Ridlon et al. 2006).

Several bacterial species in the genus *Clostridium* have been isolated and confirmed to convert primary bile acids into secondary bile acids, a process termed bile acids 7α-dehydroxylation. There previously proposed a multistep biochemical pathway for bile acids 7α-dehydroxylation. The current model of bile acid 7α-dehydroxylation suggests that free primary bile acids are actively transported into the bacterial cell by a proton-dependent bile acids transporter encoded by the baiG gene (Mallonee and Hylemon 1996). Once inside, the primary bile acids is ligated to an ATP-dependent CoA, adjusted by the baiB gene product (Mallonee et al. 1992). The baiA gene encodes 3α-HSDH, which is specific for primary bile acid CoA conjugates (Mallonee et al. 1995). It is recently reported that the baiCD and baiH gene products encode stereospecific 3-dehydro-4-bile acid oxidoreductases, which can recognize 7α-hydroxy bile acids (CA, CDCA) and 7β-hydroxy bile acids [UDCA, 3α,7α-dihydro-5β-cholan-24-oic acid], respectively (Kang et al. 2008). The rate-limiting and irreversible step in this pathway is 7α-dehydration that catalyzed by bile acid 7α-dehydratase, which is encoded by the baiE gene (Dawson et al. 1996). The 3-dehydro-4,6-bile acid intermediate is then sequentially reduced and exported from the cell. However, genes in the “reductive arm” of the pathway have yet to be identified. Previously, the baiF gene product hydrolyzes bile acid CoA conjugates (Ye et al. 1999). Moreover, the product of gene based on amino acid sequence comparisons may be a bile acid CoA transferase. Further, there exists strong evidence that the baiF gene encodes a bile acid CoA transferase with broad bile acid substrate specificity (Heider 2001). In a word, the discovery and characterization demonstrate that bile acid 7α-dehydroxylation is a multistep pathway and suggest the presence of multiple bai genes.

## Bile Acid Synthesis, Transporters, and Regulatory Nuclear Receptors in the Enterohepatic Circulation

Bile acid (BA) transporters are indispensable for maintaining the enterohepatic circulation of bile acids. Maintenance of the enterohepatic BAs circulation is vital for several liver and gastrointestinal functions including bile flow, solubilization and excretion of cholesterol, clearance of toxic molecules, intestinal absorption of lipophilic nutrients, as well as metabolic and antimicrobial effects (Hofmann 2007). In the body, BAs are actively taken up from the lumen of the terminal ileum efficiently, which is carried in by the apical sodium-dependent BA transporter (ASBT, gene symbol SLC10A2), meanwhile leaving only approximately 5 % (or approximately 0.5 g/d) in the lumen (Dietschy and Turley 2002). In contrast, this fraction is in part passively absorbed in the colon, a process facilitated by bacterial transformation (see above), and the others extruded with faces.

Bile acids homeostasis in the enterohepatic circulation is controlled by genes of nuclear receptors (NRs). Except NRs as intracellular BA sensors, some cells also contain BA receptors at the cell surface including a G-protein-coupled receptor (TGR5/M-BAR/GPBAR1) (Maruyama et al. 2002) and the epidermal growth factor receptor (Rao et al. 2002). Under physiological conditions, these regulatory networks preserve the enterohepatic BAs circulation and limit intracellular levels of potentially toxic BAs. Hence it is necessary to maintain proper BAs homeostasis regulated by transporters via NRs. Furthermore, hereditary and acquired defects of BA transporters are involved in the pathogenesis of several hepatobiliary disorders such as cholestasis and gallstones.

### The Enterohepatic Circulation of Bile Acid

#### Bile Acid Synthesis in Liver

Bile acids are synthesized from cholesterol by either a classical pathway or an alternative pathway resulting in formation of CA or chenodeoxycholic acid (CDCA). In the classical pathway, the cholesterol undergoes a series of hydroxylations catalyzed by cytochrome P450 enzyme CYP7A1, CYP8B1 (Eggertsen et al. 1996), and CYP27 (Cali and Russell 1991). In the alternative pathway, 7α-hydroxylation is preceded by the formation of several different oxysterols. Oxysterols is hydroxylated by CYP7B1 and CYP39A1 (Li-Hawkins 2000; Schwarz 1997). Moreover oxysterols are also substrates of CYP7A1 (Norlin et al. 2000) and 7α-hydroxylation of oxysterols blocks their ability to inhibit sterol regulatory element-binding protein (SREBP) (Schroepfer 2000). Thus, 7α-hydroxylation of oxysterols can markedly influence lipid metabolism through increasing the expression of genes normally regulated by SREBP. Meanwhile the low-density lipoprotein receptor (LDLR) (Dueland 1992) also regulates the expression of SREBP-regulated genes.

#### Hepatocellular Bile Salt Excretion Canalicular Export Systems

At the canalicular membrane, highly specialized canalicular transporters mediate excretion of the individual components of bile such as BAs, phospholipids, and cholesterol (Trauner 2003). The bile salt export pump (nomenclature BSEP, ABCB11 or sister of p-glycoprotein (Spgp)) is the major canalicular BAs efflux system (Gerloff et al. 1998). Importantly, the existence of other transporters is proved by Makishima (1999) and Stieger et al. (2011) by means of BSEP konckout mouse. BSEP expression and activity are tightly controlled at transcriptional and post-transcriptional levels. Farnesoid X receptor (FXR) upregulates BSEP expression (recently reviewed in Stieger 2011), while BSEP is downregulated by inflammatory, injury, and estrogen, for example obstructive cholestasis (Wagner et al. 2010). The canalicular membrane also contains transport systems mediating excretion of biliary phospholipids (nomenclature MDR3, MDR2 in rodents or ABCB4) and cholesterol (ABCG5/8), which are tightly coupled with BAs excretion (Lo et al. 2008). Another ABC transporter involved in cholesterol transport is ATP-binding cassette, sub-family A 1 (ABCA1), which mediates cellular cholesterol efflux from peripheral macrophages but also expressed at the basolateral surface of hepatocytes and Caco2 cells (Ohama et al. 2002; Neufeld et al. 2002). There are other canalicular transport systems that are less important for BAs transport. For instance multidrug resistance-associated protein 2 (MRP2/ABCC2) mainly excretes bilirubin–glucuronides and glutathione conjugates, but also transports divalent sulfo-conjugated BAs into the bile.

#### Alternative Basolateral Efflux Systems in Hepatocytes

When hepatocellular BAs overload, BAs can also be transported back to the sinusoidal blood to protect the liver and then undergo elimination through the urine. To provide less toxic and higher affinity substrates for the basolateral BAs export systems, BAs is associated with phase I and II detoxification (Zollner 2006). Basolateral efflux systems contain the multidrug resistance-associated proteins MRP3 (ABCC3), MRP4 (ABCC4), and the heterodimeric organic solute transporter (OSTα/OSTβ). Analyses of the role of MRP3 in liver with respect to endobiotics have focused on glucuronides and monoanionic bile acids, which are well-established transport substrates of the pump. MRP3 mice made cholestatic by bile duct ligation have lower serum levels of bilirubin glucuronide, a conjugate formed in the liver, compared to cholestatic wildtype mice.

#### Cholangiocytes and Bile Acid Transport

Bile duct epithelial cells (cholangiocytes) are important modifiers of bile formation by promoting bicarbonate excretion and the bile ducts act as drainage system for BAs flowing to the intestine. After hepatocytes secrete bile salts into the bile canaliculus, primary hepatic bile acids are modified during its passage through the biliary tree by organic anion and electrolyte transport proteins expressed in biliary epithelial cells. In contrast, conjugated BAs require active transport into cholangiocytes via an apical sodium-dependent BA transporter (ASBT/SLC10A2), which is identical to the transport system in the ileum (see below) (Lazaridis et al. 1997). BAs are exported into the adjacent peribiliary capillary plexus via OSTα/β, MRP3, and possibly a truncated version of ASBT (tABST) (recently reviewed in (Claudel et al. 2011)). The physiological role of bile salts uptake by cholangiocytes probably pertains to the regulatory effect of bile salts on intracellular signaling mechanisms including cholangiocellular mucin and bicarbonate secretion (Hirata 2001; Jungst and Sreejayan 2007).

#### Intestinal Bile Acid Transporters

BAs are mainly actively taken up in the terminal ileum via ASBT, apart from a relatively small proportion of passive uptake in the proximal small intestine and colon (Shneider 2001; Dawson et al. 2003). Interestingly, enterocytes, cholangiocytes, and renal tubular cells share several BA transport systems including ASBT (see above) (Zollner 2006). After reabsorption, BAs are bound to the cytosolic ileal BA binding protein IBABP (also known as ileal lipid binding protein (ILBP) and fatty acid binding protein 6, FABP6) and exported into the portal blood via OSTα/OSTβ (Dawson et al. 2009). Bile acids escape ileal reabsorption and are transformed by intestinal flora in the colon, where the secondary BAs take up through efflux systems (e.g., OSTα/OSTβ) (Zollner 2006).

#### Hepatocellular Uptake of Bile Salts

The final step in the enterohepatic circulation of bile salts is the extraction from portal blood plasma by hepatocytes, whereas a much lesser amount from the hepatic artery, and are efficiently removed during their first passage through the hepatic sinusoids by hepatocellular BA uptake systems (Kullak-Ublick et al. 2004), involving a sodium-dependent sodium/taurocholate co-transporting polypeptide (NTCP/SLC10A1) and a family of sodium-independent multispecific organic anion transporters (OATPs/SLCOs) (Kullak-Ublick et al. 1994; Hagenbuch 1994). This process is described in detail in several recent review articles (Trauner 2003). Bile salts circulate in plasma are tightly bound to albumin and lipoproteins such as high-density lipoprotein (Wolkoff and Cohen 2003). NTCP accounts for the bulk (about 90 %) of BA uptake and is the first cloned BA transporter (Hagenbuch 1994). Its regulation under physiological and pathological conditions is therefore well understood thus serving as a paradigmatic model to understand transporter regulation. NTCP expression is controlled by BAs, hormones such as estrogen and prolactin, as well as pro-inflammatory cytokines (recently reviewed in Wagner et al. 2010).

Next to the sodium-dependent uptake system NTCP, the organic anion transporting polypeptides 1B1 (OATP1B1, encoded by the SLCO1B1 gene), OATP1B3 (SLCO1B3), and OATP2B1 (SLCO2B1) are major uptake transporters on the sinusoidal membrane of human hepatocytes. They mediate the influx of endogenous compounds such as bile salts, bilirubin glucuronides, thyroid hormones and steroid hormone metabolites, and clinically frequently used drugs like statins, HIV protease inhibitors, and the anti-cancer agents irinotecan or methotrexate (Hagenbuch 2004; Giacomini et al. 2010; Niemi 2011; Nies et al. 2008).

### Transcriptional Regulation of Bile Salt Transporters

Expression of bile salt transporters in enterohepatic circulation determines not only uptake and efflux systems but also key bile acid synthetic enzymes. To ensure the balance between synthesis, uptake and excretion, expression of bile salt transporters is tightly regulated by NRs. NRs provide a network of negative feedback and positive feed-forward mechanisms, in order to control intracellular concentration of biliary constituents, which are often also ligands for these NRs.

#### Liver X Receptor

Liver X receptors (LXRs) were initially characterized as sterol sensors that affect cholesterol and lipid homeostasis and inflammation (Tontonoz 2003). In rodents, LXRα promotes bile acid synthesis by activating cytochrome P450 7A1 (Cyp7a1), a rate-limiting enzyme, to convert cholesterol to bile acids (Peet et al. 1998; Chiang 2004). It showed that LXR can also promote bile acid detoxification and alleviate cholestasis. LXRs are known to induce the expression of cholesterol and phospholipid efflux transporters. These include the canalicular cholesterol efflux transporters ABCG5 and ABCG8 (Repa et al. 2002), as well as ABCA1, a basolateral ABC transporter that effluxes both cholesterol and phospholipids (Chawla 2001; Groen et al. 2001; Mulligan et al. 2003). The potential effects of LXR on the homeostasis of bile acid, cholesterol, and phospholipids may play a role in the pathogenesis of cholesterol gallstone disease (CGD). LXR-mediated increases in the biliary efflux of cholesterol and phospholipids as well as a reduced biliary bile salt pool size, resulting from sensitizing mice of lithogenic diet, induced gallbladder cholesterol crystallization (Uppal et al. 2008).

#### Role of the Farnesoid X Receptor (FXR) in Controlling Bile Acid Homeostasis

FXR has a predominant role in regulating bile acid synthesis and bile salt transport. A rise in intracellular bile acid levels results in an increase in binding of bile acids to the ligand-binding domain of FXR and in transcriptional activation of its target genes. One such hepatic FXR target gene is the small heterodimer partner (SHP; NR0B2) (Goodwin 2000), an atypical member of the NR superfamily that lacks a DNA-binding domain (Seol 1996). SHP can dimerize with and inactivate both LRH1 and LXRa, resulting in a decrease in Cyp7a1 expression (Goodwin 2000; Brendel and Schoonjans 2002). Support for this model comes from studies showing that treatment of SHP impaired mice with a potent, synthetic FXR agonist (GW4064) fails to repress Cyp7a1 mRNA levels (Kerr et al. 2002).

Another pathway that regulates bile acid production is initiated after activation of FXR in enterocytes; this activation is induced through enhancing transcription and secretion of fibroblast growth factor 15 (FGF15). Subsequent binding of FGF15 to fibroblast growth factor receptor 4 (FGFR4), a transmembrane tyrosine kinase receptor localized on the hepatocyte cell surface, results in activation of the c-Jun N-terminal kinase (JNK) pathway and repression of Cyp7a1 and Cyp8b1 (Inagaki et al. 2005). A prove for the importance of FGF15 in this pathway came from the earlier observation that both Cyp7a1 expression and the bile acid pool are increased in FGFR4-lacking mice (Yu et al. 2000).

In addition to Cyp7a1 and Cyp8b1, FXR also regulates genes involved in bile acid (e.g., BSEP, MRP2) and phospholipid (e.g., human MDR3 or mouse Mdr2) secretion across the bile canalicular membrane, bile acid transport (ASBT, NTCP, IBABP, OSTα–OSTβ), and bile acid conjugation and detoxification (e.g., SULT2A1, UGT2B4, BACS, BAT) (Lee et al. 2006).These findings suggest that FXR is the primary bile acid sensor that coordinately regulates bile acid metabolism.

#### Pregnane X Receptor (PXR) in Bile Acid Metabolism

PXR regulates both the basal expression and repression of Cyp7a (Staudinger et al. 2001a; 2001b). Interestingly, PXR does not regulate Shp expression. Thus, PXR represses Cyp7a1 through a mechanism distinct from that of FXR. In addition to Cyp7a1, PXR also regulates other genes that have also been implicated in bile acid metabolism. These genes include MRP2 (Kast et al. 2002) and OATP2 (Staudinger et al. 2001b), which transport bile acids across hepatic canalicular and sinusoidal membranes, respectively (48); and, CYP3A, which hydroxylates bile acids including lithocholic acid (LCA) (Araya and Wikvall 1999). Unlike FXR, PXR is not activated efficiently by CA, chenodeoxycholic acid, or their conjugated derivatives. However, both the mouse and human PXR are activated efficiently by the secondary bile acid LCA and its 3-keto metabolite (Staudinger et al. 2001; Xie et al. 2001).

#### Role of Other Transcription Factor (VDR, CAR, PPARα, GR) in the Regulation of Bile Salt

The basolateral bile salt transporter Mrp3, which is strongly induced in obstructive cholestasis in rats and compensates for decreased Mrp2 expression, is induced by constitutive androstane receptor (CAR) (Soroka et al. 2001). CAR is a NR known to strongly activate CYP2B1 expression, and recent evidence suggests that bilirubin may induce translocation of CAR from the cytosol to the nucleus, suggesting a regulatory role of CAR in cholestatic liver disease (Cherrington et al. 2002; Xiong and Yoshinari 2002).

Vitamin D receptor (VDR) binds 1α,25-(OH)2-vitamin D3 with high affinity and mediates classic calcitriol effects such as regulation of calcium and phosphate homeostasis. Krasowski et al. (2011, 2008) suggest that only VDRs from animals that predominantly use 5β-bile acids are activated by bile acids, possibly as an adaptive response to limit the toxicity of secondary bile acids generated in the intestinal tract.

The apical sodium-dependent bile salt transporter ASBT and the hepatocyte canalicular phospholipid flippase Mdr2/MDR3 (ABCB4) are activated by PPARα (Jung and Fried 2002; Kok 2003). A key function of PPARα is the regulation of genes involved in various steps of fatty acid metabolism (Desvergne and Wahli 1999) and PPARα ligands include fatty acids and fibrate drugs. Fibrate drugs are ligands of PPARα and an important adverse effect of fibrate treatment is the increased risk of cholesterol gallstone formation (see below).

The glucocorticoid receptor (GR) is a nuclear steroid hormone receptor that is activated by nanomolar concentrations of glucocorticoids. The human ASBT gene has been proved to be transactivated by the GR and an induction of ASBT by glucocorticoids could be beneficial in patients with Crohn’s disease who exhibit reduced ASBT expression (Jung 2004). The NTCP promoter is activated by GR in a ligand-dependent manner, similarly to the ASBT promoter. Thus, glucocorticoids may coordinately regulate the major bile acid uptake systems in human liver and intestine, while providing a negative feedback mechanism for bile acid uptake in human hepatocytes.

## Enterohepatic Circulation and Gallstone

In theory, each factor that disequilibrates the enterohepatic circulation will lead to the formation of gallstones. Nonetheless, there still remain many unknown aspects when it comes to regulation of the bile acid homeostasis in the enterohepatic circulation. In the past few decades, it’s widely accepted that the most important prerequisites for gallstone formation are hypersecretion of biliary cholesterol and cholesterol supersaturation of the bile. Meanwhile, gallstone also exhibits abnormality of bile acid, reduction of gallbladder motility, prolongation of intestinal transit time. In the following, we will expose the cause of gallstones in detail.

### Role of Bacteria in Gallstone Disease

Secondary bile acids are synthesized in the human colon from the bacterial 7alpha-dehydroxylation of primary bile acids. Increased levels of secondary bile acids have been correlated with an increased risk of CGD. Moreover, increased CA-7alpha-dehydroxylation activity of the intestinal microflora may be an important factor for cholesterol gallstone formation or growth in gallstone patients. High levels of CA 7a-dehydroxylating fecal bacteria have been correlated with increased amounts of DCA in bile of a subset of cholesterol gallstone patients. Treatment of these cholesterol gallstone patients (high DCA group) with antibiotics significantly decreased the levels of fecal CA 7a-dehydroxylating bacteria, DCA in bile, and the cholesterol saturation index in bile (Berr 1996). The optimal reaction conditions of cholylglycine hydrolase and 7α-dehydroxylase are measured in fresh caecal samples, obtained by aspiration during clinically-indicated colonoscopy. This is an essential first step to further studies of intestinal bacterial enzymes in the pathogenesis of CGD (Thomas et al. 1997). UDCA, which can be used as an therapeutic agent for the non-surgical dissolution of gallstones as well as for the treatment of primary sclerosing cholangitis and primary biliary cirrhosis (Beuers et al. 1992). The key enzymatic steps in the synthesis of UDCA are the reduction of dehydrocholic acid (DHCA) to 12-keto-ursodeoxycholic acid. Recently, it is observed that 7β-hydroxysteroid dehydrogenase (7β-HSDH) from *Collinsella aerofaciens*, 3α-hydroxysteroid dehydrogenase (3α-HSDH) from *Comamonas testosteroni* with glucose dehydrogenase(GDH) from *Bacillus subtilis* for cofactor regeneration, which was able to completely convert 100 mM DHCA to >99.5 mM 12-keto-UDCA within 4.5 h in a simple batch process on a liter scale (Sun et al. 2013).

Acromegalic patients have slow colonic transit, increased rates of deoxycholic acid formation, and an increased prevalence of cholesterol gall stones, especially during long-term octreotide treatment. The increasing deoxycholic acid formation seen in acromegalics during octreotide treatment is due not only to the greater numbers of facal anaerobes but also to increased activity of the rate-limiting enzyme pathway (7alpha-dehydroxylation) converting cholic acid to deoxycholic acid (Thomas et al. 2005). It is found that gallstone patients had >42-fold (*p* < 0.01) higher levels of 7alpha-dehydroxylating bacteria than patients who had not developed gallstones. And all strains of 7alpha-dehydroxylating bacteria isolated from gallstone patients appear to belong to the genus *Clostridium* (Wells et al. 2000). Moreover, antibiotic treatment decreases facal 7α-dehydroxylation activity, and lowers biliary deoxycholate and cholesterol concentration (Berr 1996).

Bacterial DNA sequences are usually present in mixed cholesterol (to 95 % cholesterol content), brown pigment, and common bile duct, but rarely in pure cholesterol gallstones (Lee et al. 1999). Stewart and co-authors (2007, 2007) have readily cultured bacteria from cholesterol stones with pigment centers. Bacteria sequestered in cholesterol stones cause fewer infectious than pigment stone bacteria. Possibly because of their isolation, cholesterol stone bacteria were less often present in bile and blood, induced less immunoglobulin G, were less often killed by a patient’s serum. Bacteria-laden gallstones are biofilms whose characteristics influence illness severity. Factors (beta-glucuronidase/phospholipase) creating colonization surface facilitated bacteremia and severe infections. But at the same time, abundant slime production not only facilitates colonization, but also inhibits detachment and cholangiovenous reflux. What is more, germ-free rederivation rendered mice more susceptible to cholesterol gallstone formation. This susceptibility appeared to be largely due to alterations in gallbladder size and gallbladder wall inflammation (Fremont-Rahl et al. 2013).

### Role of Intestine in Gallstone Disease

In gallstone patients, both small intestinal and whole gut transit times are prolonged compared to normal controls. Therefore, we can conjecture that impaired intestinal motility might promote gallstone formation. One possible link between impaired intestinal motility and lithogenic bile could be secondary hydrophobic bile salt deoxycholate. We can find the evidence from studies in humans and mice. For example, it is reported that gallstone patients have longer large bowel transit times, more total and gram-positive anaerobes and more 7α-dehydroxylating activity in the caecum than normal subjects (Thomas et al. 2000). Further, treatment with octreotide that is known for the risk factor of CGD, which not only increases biliary deoxycholate concentration but also prolongs colonic transit time (Thomas et al. 2005).

It is also believed that ileal disease, bypass, or resection represent a major risk factor for gallstones, according to study on prairie dogs that underwent ileal resection or Crohn’s disease, whose cholesterol saturation indexes of gallbladder bile remained essentially unchanged, whereas pigment gallstones formed frequently (Jung 2004; Pitt et al. 1984). The role of chronic intestinal infection as a potential factor in cholesterol gallstone pathogenesis has been proposed. Distal intestinal infection with a variety of enterohepatic Helicobacter species, but not Helicobacter pylori, are essential to nucleate cholesterol supersaturated bile in a well-established murine model of cholesterol gallstone formation (Maurer et al. 2006; Maurer et al. 2005). Bile salt malabsorption is another factor of gallstone. For example, excess dietary carbohydrates are known to cause bile salt malabsorption. Bile salt malabsorption has been induced in mice and hamsters by a diet rich in nondigestible starch and β-cyclodextrin (Abadie et al. 1994). Similar results have been found with diets replete in refined sugars: high sucrose diets increase fecal bile salt loss, possibly because of induction of more rapid intestinal transit (Kruis et al. 1991). Meanwhile, chronic alcohol ingestion by laboratory animals leads to bile salt malabsorption due to morphological and functional alterations of the small intestinal mucosa, which may compromise Na+ -coupled bile salt transport (Zucoloto and Muccilo 1985). Recent experimental studies on mice with defects in the cystic fibrosis transmembrane conductance regulator gene (CFTR) have shown that bile salt malabsorption also occurs in these animal models, which may be caused by the disorder of mucin, and leads to gallstone (Debray et al. 2012). Moreover, prolonged TPN may also cause ileal atrophy and downregulation of ileal ASBT, complicating the scenario with added bile salt malabsorption (Matsumura et al. 1993).

### Role of Gallbladder in Gallstone Disease

Impaired postprandial gallbladder emptying, often present in cholesterol gallstone patients, may prolong residence of bile in the gallbladder, thus allowing more time for nucleation of cholesterol crystals from supersaturated bile and their growth/aggregation into macroscopic stones. Although impaired motility is generally secondary to biliary cholesterol supersaturation, it may still facilitate the process of gallstone formation. Gallbladder motility is often impaired in many high-risk situations for gallstone formation, such as pregnancy, obesity and rapid weight loss in obese patients, diabetes mellitus, and total parenteral nutrition (van Erpecum et al. 2000). Meal-induced release of cholecystokinin (CCK) from the duodenum is the principal factor driving gallbladder smooth muscle contraction, accounting for 70–80 % of the decrease of fasting gallbladder volume. The CCK-1-receptors-deficient mice have larger gallbladder volumes (predisposing to bile stasis), significant retardation of small intestinal transit times (resulting in increased cholesterol absorption), and increased biliary cholesterol secretion rates. And the mice result in a significantly higher prevalence of cholesterol gallstones for the absence of CCK-induced contraction (Wang et al. 2004). A primary role for gallbladder motility in gallstone formation is also indirectly supported by the observation that daily CCK injection during total parenteral nutrition or inclusion of dietary fat to enhance CCK release during rapid weight loss restores gallbladder contractility and can prevent gallstone formation (Gebhard et al. 1996; Sitzmann et al. 1990).

Gallbladder wall inflammation may also be critical in gallstone formation. The gallbladder wall is exposed to detergent bile salts, unesterified cholesterol and bacteria, which all could induce inflammation. The murine infected with *Helicobacter* spp and fed a lithogenic diet develops cholesterol gallstone at 80 % prevalence by 8 weeks compared with approximately 10 % in uninfected controls. This result indicates that *Helicobacter* spp play a significant role in the pathophysiology of cholesterol gallstone formation in mice and perhaps humans (Maurer et al. 2005). Subsequent studies confirm that T cells are critical in murine cholesterol cholelithogenesis. Wild-type mice develop significantly more cholesterol gallstones than congenic immunodeficient Rag2(−/−) (Rag) mice. Meanwhile T lymphocytes to Rag2(−/−) mice increased stone prevalence markedly (Maurer et al. 2007). Although various helicobacter species have been detected in human gallbladders and bile, and antibodies to helicobacter hepaticus were found at increased frequency in gallstone patients, the role of helicobacter in human gallstone pathogenesis remains to be defined.

### Role of Bile Acids Transporters in Gallstone Disease

Bile acids transporters play a important role in pathogenesis of gallstone. As it is known to us, enterocytes, cholangiocytes, and renal tubular cells all contain apical sodium-dependent bile acid transporter (ASBT) which is one of BAs transport systems. Interestingly, female who are deficient of the ASBT and ILBP in the cholesterol, low BAs or phospholipid concentrations, as well as BAs and phospholipid species, deter small intestine result in promoting gallstone formation (Bergheim et al. 2006). It observes that an impaired function of OSTα–OSTβ may lead to low ileal bile acid reabsorption and an altered bile acid pool composition and therefore may contribute to the formation of gallstones in non-obese patients (Renner et al. 2008). A decreased expression of the ileal ASBT gene SLC10A2 develops the formation of gallstone, and Comprehensive statistical analysis provides strong evidence that allele of rs9514089 is a genetic determinant especially in male non-obese gallstone carriers (Renner et al. 2009). What is more, the decreased hepatic NPC1L1 levels may leads to the cholesterol supersaturation because of the malabsorption of biliary cholesterol in the liver in Chinese female gallstone patients (Cui et al. 2010).

The process of nascent bile formation is maintained by an elaborate network of ATP-binding cassette (ABC) transporters in the hepatocyte canalicular membrane which enable biliary secretion of cholesterol, bile salts, and phospholipids. Higher hepatic messenger RNA expression of ABCG5 and ABCG8 correlates positively with higher biliary cholesterol levels (Yu 2002). The variants ABCG8(D19H) are proved to be a susceptibility factor for human gallstone disease via a genome-wide association scan (Buch et al. 2007). Recently, mutation detection and genotyping yield two chololithiasis-associated variants: ABCG5-R50C and ABCG8-D19H. Moreover, the ABCG8-19H variant has a high transport capacity, which was also superior in nested logistic regression models in German, Chilean, and Chinese patient samples (von Kampen et al. 2013). Henkel and Wei (2005) observes that overexpress BESP (ABCB11) in mice fed a lithogenic diet have an increased rate of cholesterol crystal and gallstone formation. This was associated with an increase in both the hydrophobic bile salt and cholesterol content of gallbladder bile. Analysing the entire MDR3 (ABCB4) gene coding sequences represents that MDR3 (ABCB4) gene mutations are a major genetic risk factor in a symptomatic and recurring form of cholelithiasis in young adults (Rosmorduc and Hermelin 2003). Nevertheless, recent data from sib pairs with gallstones and control do not support a link between ABCB4 and ABCB11 polymorphisms and gallstone formation in the large majority of patients (Acalovschi et al. 2009).

The down-regulation of NTCP1 (SLC10A1) protein expression might protect hepatocytes from high intracellular bile salt loads in the lithogenic diet mice (Muller et al. 2002). Organic anion transport protein 1B1 (OATP1B1) (encoded by SLCO1B1) is a major transporter protein for bile salt uptake in enterohepatic circulation of bile salts. The frequency CA genotype and A allele of Exon4 C > A polymorphism was higher in gallstones patients (12.4 and 6.2 %) as compared to controls (5.2 and 2.6 %). These results confirm the increasing risk of gallstone disease in North Indian population (Srivastava et al. 2011).

### Role of Nuclear Receptors in Gallstone Disease

FXR is a member of the NR superfamily and regulates hepatic expression of BESP (ABCB11) and MDR3 (MDR2/ABCB4), thus affecting amounts of solibilizing bile salts and phospholipids in bile. FXR deficient mice are highly susceptible to gallstone formation, which treating with a synthetic FXR agonist can prevent gallstone disease. These results maybe relate to the low amounts of biliary bile salts and phospholipids, which are regulated by BESP and MDR3 (Moschetta 2004). What is more, research suggests that FXR and the heterodimer ABCG5/ABCG8 are possible determinants of cholesterol gallstone formation in mice via quantitative trait locus analysis (Wittenburg 2003). The loss of the ASBT and ILBP)in female normal weight gallstone carriers is coupled with a reduction of protein levels of hepatic nuclear factor 1alpha and FXR (Bergheim et al. 2006). The absence of β-Klotho would be predicted to disrupt the FXR–FGF15/19-mediated gut liver signaling, the most relevant pathway for FXR-dependent CYP7A1 down-regulation (Franz et al. 2001), leading to increased CYP7A1 levels. Thus, inhibition of βKlotho could improve current (i.e., UDCA) and potential future therapies (i.e., synthetic FXR agonists) for gallstone disease by increasing cholesterol breakdown via increasing CYP7A1, which is typically reduced in treatment with FXR agonists.

We have illustrated that LXR regulates expression of ABCG5/G8 cholesterol transport protein. The current study has revealed a novel lithogenic role of LXR as well as a functional interplay between LXR and LDLR in gallbladder cholesterol crystallization and possibly CGD in the murine model. Furtherly, in a small group of Chinese non-obese normolipidemic gallstone patients, the upregulation of ABCG5/ABCG8 in gallstone patients is mediated by increased LXRalpha possibly, may contribute to the cholesterol supersaturation of bile and the formation of gallstone (Jiang et al. 2008). In addition, LXRβ and PPARδ coordinate Niemann-Pick C1-like L1(NPC1L1/ABCA1)-dependent vectorial cholesterol flux from bile through cholangiocytes and manipulation of these processes may influence bile composition in murine with gallstone (Xia et al. 2012).

PXR knock-out mice associated with reduced expression of cholesterol 7alpha-hydroxylase present a decrease in biliary concentrations of bile salts and phospholipids and an increase in the cholesterol saturation index and formation of cholesterol crystals (He et al. 2011). Fasted mice with hepatocyte-specific GR knockdown have smaller gallbladder BA content and are more susceptible to developing cholesterol gallstones when fed a cholesterol-rich diet. Hepatic GR deficiency reduces the expression of the major hepatocyte basolateral BA transporter, Na(+)-taurocholate transport protein (NTCP/SLC10A1), which affected dietary fat absorption and brown adipose tissue activation (Rose et al. 2011). Role of VDR in BESP repression via direct VDR–FXR interaction has been postulated in vitro. And VDR is able to induce mouse MRP3 expression in intestine (Honjo et al. 2006). CAR appears to play a central role in regulating bile acid sulfation, since it is proposed to regulate bile acid sulfation and subsequent basolateral export via CAR-induced over-expression of the basolateral export pump MRP4, which transports steroid sulfates (McCarthy et al. 2005). But, there is not any direct research between VDR or CAR and the formation of gallstone. Further research is needed on the role of NRs in gallstone pathogenesis and the therapeutic potential of the potent NR agonists currently available. The review shows the latest information as far as we know it, but there is still much work remains to be done to identify the causal relation between enterohepatic circulation and gallstone.

## Abbreviations

BA: Bile acid
BSH: Bile salt hydrolase
HSDH: Bile acid hydroxysteroid dehydrogenase
CA: Cholic acid
LCA: Lithocholic acid
DCA: Deoxycholic acid
CDCA: Chenodeoxycholic acid
UDCA: Ursodeoxycholic acid
DHCA: Dehydrocholic acid
CYP: Cytochrome P450 enzyme
BSEP: Bile salt export pump
ABCA: ATP-binding cassette, sub-family A
ABCB: ATP-binding cassette, sub-family B
MDR3: Multidrug Resistance Transporter 3
MDR2: Multidrug Resistance Transporter 2
ABCG5/8: ATP-binding cassette, subfamily G, member 5/8
MRP: Multidrug resistance-associated proteins
NTCP: Na+ taurocholate cotransporting polypeptide
OATP: Organic anion transporting polypeptide
SLCOs: Solute carrier family
OST: Organic solute transporter
ASBT: Apical sodium bile acid transporter
LXR: Liver X receptor
FXR: Farnesoid X receptor
PXR: Pregnane X receptor
VDR: Vitamin D receptor
CAR: Constitutive androstane receptor
GR: Glucocorticoid receptor
PPARα: Peroxisome proliferator-activated receptor α
SHP: Small heterodimer partner
TNFα: Tumor necrosis factor alpha
HNF1α: Hepatic nuclear factor 1α
ILBP: Ileal lipid binding protein
SULT2A1: Sulfotransferases2A1
UGT2B4: Uridine dipho-sphate glucuronosyltransferase 2 family, polypeptide B4
BACS: Bile acid-CoA synthetase
BAT: Bile acid-CoA amino acid N-acetyltransferase
CCK: Cholecystokinin
FGF: Fibroblast growth factor
NPC1L1: Niemann-Pick C1-like L1
